# Bioinformatics and Experimental Analyses Reveal NFIC as an Upstream Transcriptional Regulator for Ischemic Cardiomyopathy

**DOI:** 10.3390/genes13061051

**Published:** 2022-06-13

**Authors:** Yang Ye, Qiao Jin, Qian Gong, Aoqi Li, Minghao Sun, Sibo Jiang, Yulan Jin, Zhe Zhang, Jin He, Lenan Zhuang

**Affiliations:** 1 Key Laboratory of Cardiovascular Intervention and Regenerative Medicine of Zhejiang Province, Department of Cardiology, Sir Run Run Shaw Hospital, College of Medicine, Zhejiang University, Hangzhou 310016, China; yeyang1222@zju.edu.cn; 2 Department of Veterinary Medicine, College of Animal Sciences, Zhejiang University, Hangzhou 310058, China; 22117037@zju.edu.cn (Q.J.); 12017036@zju.edu.cn (Q.G.); 119989@zju.edu.cn (A.L.); mhsun29@zju.edu.cn (M.S.); jiangsb0219@zju.edu.cn (S.J.); jinyulan@zju.edu.cn (Y.J.); 3 Institute of Genetics and Reproduction, College of Animal Sciences, Zhejiang University, Hangzhou 310058, China; zhe_zhang@zju.edu.cn (Z.Z.); hejin@zju.edu.cn (J.H.)

**Keywords:** bioinformatics analysis, ischemic cardiomyopathy, transcriptional regulator

## Abstract

Ischemic cardiomyopathy (ICM) caused by coronary artery disease always leads to myocardial infarction and heart failure. Identification of novel transcriptional regulators in ICM is an effective method to establish new diagnostic and therapeutic strategies. In this study, we used two RNA-seq datasets and one microarray dataset from different studies, including 25 ICM and 21 non-failing control (NF) samples of human left ventricle tissues for further analysis. In total, 208 differentially expressed genes (DEGs) were found by combining two RNA-seq datasets with batch effects removed. GO and KEGG analyses of DEGs indicated that the response to wounding, positive regulation of smooth muscle contraction, chromatin, PI3K-Akt signaling pathway, and transporters pathways are involved in ICM. Simple Enrichment Analysis found that NFIC-binding motifs are enriched in promoter regions of downregulated genes. The Gene Importance Calculator further proved that *NFIC* is vital. *NFIC* and its downstream genes were verified in the validating microarray dataset. Meanwhile, in rat cardiomyocyte cell line H9C2 cells, two genes (*Tspan1* and *Hopx*) were confirmed, which decreased significantly along with knocking down *Nfic* expression. In conclusion, *NFIC* participates in the ICM process by regulating *TSPAN1* and *HOPX*. *NFIC* and its downstream genes may be marker genes and potential diagnostic and therapeutic targets for ICM.

## 1. Introduction

Ischemic heart disease is among the most common causes of morbidity and mortality worldwide [1]. The histological damage associated with scar tissue and the nonreciprocal loss of cardiomyocytes eventually leads to functional depression–heart failure [2]. The currently available therapeutic strategies such as cardiovascular drugs, interventional surgery, and coronary artery bypass grafting, which improve symptoms, cannot fundamentally reverse the condition. Thus, new therapeutic strategies need to be established.

Previous studies have reported that important transcription factors have considerable influence in ICM on account of their special binding motif. For example, the delivery of transcription factor GATA4 locally to the infarct border zone demonstrates increased cardiac function and less negative remodeling [3]. Another transcription factor, Foxo3a, activates the expression of the apoptosis repressor with a caspase recruitment domain by directly binding to its promoter; thus, maintaining calcium homeostasis and inhibiting cardiac apoptosis caused by ICM [4]. NFIC is a transcription factor, a member of the NFI family. The initial study has found that the NFI family is a transcription factor family necessary for adenovirus DNA replication [5], which consists of four members in humans and most vertebrates: NFIA, NFIB, NFIC, and NFIX [6]. They share a highly conserved DNA-binding motif at their N-termini, and therefore bind to a common DNA sequence [7,8]. Studies have shown that NFIA, NFIB, and NFIX play important roles in different organs [9,10,11]. In previous studies, NFIC is regarded as a key regulator of tooth development. NFIC deficiency severely disrupts odontoblast differentiation, leading to the formation of aberrant odontoblasts in the early stage of root formation [12]. Accumulative evidence has also revealed that NFIC is involved in regulating adipocyte and osteoblast differentiation [13,14]. However, the role of NFIC in the heart is unclear.

The rapidly developing integrated bioinformatics analyses have become a powerful tool for predicting disease-associated genes, disease subtypes, and disease treatment. Due to different criteria regarding experimental conditions and sample selection between different datasets, the gene expression levels always show diversity in different datasets. Meta-analysis has been widely used to overcome inconsistent findings among different studies [15,16]. A study identified two potential marker genes (*CDC5L* and *DDX46*) of pulmonary arterial hypertension by meta-analysis and animal model validation [17]. Three upstream transcriptional regulators and multiple novel dysregulated genes involved in the development of ischemic cardiomyopathy and its associated cardiovascular diseases were found by RNA-seq meta-analysis [18]. Transcription factors that play important roles in diseases are usually found by animal models and experimental methods, while bioinformatics analyses are also effective ways to search motifs.

In our study, we combined two RNA-seq datasets with the batch effects removed. In total, 118 downregulated and 90 upregulated genes were found and Kyoto Encyclopedia of Genes (KEGG) and Gene Ontology (GO) analyses were also performed to investigate the underlying mechanisms. Simple Enrichment Analysis indicated that NFIC-binding motifs are enriched in promoter regions of downregulated genes in ICM and the essentiality of *NFIC* was further proved with the Gene Importance Calculator. *NFIC* and its downstream genes were verified in validating the microarray dataset and two of them were confirmed in *Nfic* knockdown H9C2 cells. Our study may point to potential targets for the treatment of ICM.

## 2. Materials and Methods

### 2.1. Data Selection

**Figure 1:**
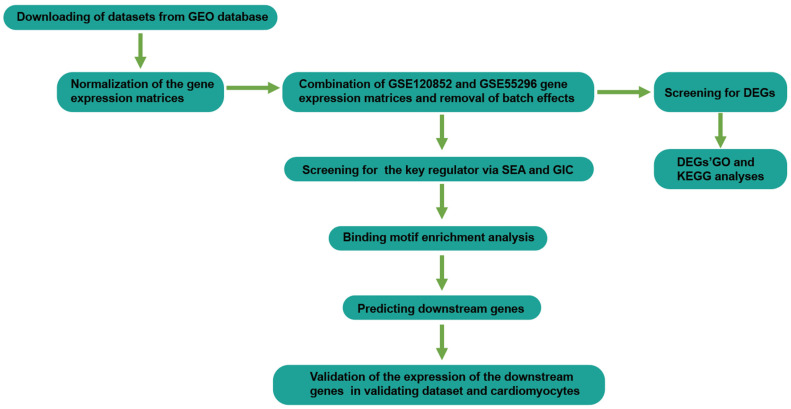
Workflow of the study design.

The RNA-seq datasets GSE120852, GSE55296 were downloaded from Gene Expression Omnibus (GEO) (http://www.ncbi.nlm.nih.gov/geo/ (accessed on 25 October 2021)). GSE120852 includes heart tissues from non-failing controls (*n* = 5) and ICM patients (*n* = 5); GSE55296 includes heart tissues from non-failing controls (*n* = 10) and ICM patients (*n* = 13). We downloaded the expression data of 18 ICM heart tissues and 15 non-failing controls from GSE120852 and GSE55296 expression profiling for further analysis. The microarray dataset GSE1869 includes heart tissues from non-failing controls (*n* = 6) and ICM patients (*n* = 7). We downloaded the Series Matrix File for further verification. Detailed information about the datasets can be found in Tables S1–S3. The workflow for bioinformatics analysis in our study is illustrated (Figure 1).

### 2.2. Data Preprocessing and DEGs Screening

A pipeline of HISAT2 [19], Samtools [20], and featureCounts [21] was used for aligning the trimmed reads to the human reference genome (GRCh38) and quantifying gene expression. Only uniquely mapped reads were used for expression quantification. The GSE120852 and GSE55296 datasets were merged with batch effects removed using the “limma” package [22]. According to the workflow, the merged dataset served as a training dataset to identify important genes while the GSE1869 dataset was used for assessment. The effect of inter-sample correction was demonstrated using PCA plots which were performed on the training matrices before and after the removal of the inter-batch effect with the “BioLadder” (https://www.bioladder.cn/web/ (accessed on 1 March 2022)). DEGs were screened using the “DESeq2” package [23] in R software (version 4.1.2) with the cutoff indicated in figure legends.

### 2.3. GO and KEGG Functional Enrichment Analyses

DAVID Bioinformatic Resources (https://david.ncifcrf.gov/ (accessed on 22 November 2021)) [24] and Weighted Enrichment Analysis Tools (WEAT, http://www.cuilab.cn/weat/ (accessed on 22 November 2021)) [25] were used to analyze downregulated genes identified by meta-analysis. In WEAT analysis, we selected the heart gene expression in Expression-GTEx_smts as the gene essentiality score and the Scaling Factor was 3.

### 2.4. Transcription Factor Binding Site Analysis

Genes that were significantly downregulated in ICM compared with NF were subjected to Transcription Factor Binding Site (TFBS) enrichment analysis using the Simple Enrichment Analysis (https://meme-suite.org/meme/tools/sea (accessed on 16 November 2021)) [26]. Predicted TFBS were identified in gene regions 5000 bp upstream of the transcription start site (TSS) for all downregulated genes. The promoter regions were exported by “biomaRt” package. NFIC motif profiles were downloaded from JASPAR TFBS database (https://jaspar.genereg.net/ (accessed on 16 November 2021)) [27].

### 2.5. Construction of Lentiviral Vectors and Cell Culture

To knockdown NFIC expression, *Nfic*-specific short hairpin RNA (shRNA)-expressing constructs (shNfic) were designed. The shRNA sequences targeting *Nfic* are listed in Table S7. The shRNA was cloned into pLKO.1 vector and empty vector was used as the control. pMD2.G and psPAX2 were transfected together with the pLKO.1 vector as the helper vectors. The ratio of the pLKO.1 vector, psPAX2, and PMD2.G is 4:3:1. They were transfected into HEK293T cells using Genetwin transfection reagent. H9C2s were purchased from China Nation Collection of Authenticated Cell Cultures. Cells were cultured in Dulbecco’s modified Eagle’s medium (DMEM) (Hyclone, Logan, UT, USA) supplemented with 10% fetal bovine serum (FBS) (ExCell, Suzhou, China), 100 IU/mL penicillin, and 100 μg/mL streptomycin (GENOM, Hangzhou, China). These cells were cultured at 37 °C in a humidified incubator under 5% CO_2_. Cultured H9C2s were infected with the indicated lentiviral vectors after three cell passages. After 24 h, the culture medium was replaced by DMEM supplemented with 10% FBS. The transduced cells were then supplied with 2 μg/mL puromycin 2 days after transduction to clear the nontransduced cells.

### 2.6. Quantitative Real-Time PCR (qRT-PCR)

Total RNA was extracted from the cultured cells by using TRIZol reagent (Tsingke, Beijing, China). The cDNA was synthesized with 2 μg of RNA using the 4×EZscript Reverse Transcription Mix II Kit (EZBioscience, Roseville, MN, USA). Then, each 96-well plate well was mixed with 6.5 μL of cDNA (diluted at 1:50), 7.5 μL of qPCR SYBR Mix (Tsingke, Beijing, China), and 1 μL of primers (10 mM). The primers used in the study are listed in Table S6.

### 2.7. Western Blotting

Cells were lysed in Loading Buffer 1× (50 mM Tris-HCl pH 6.8, 2% SDS, 10% glycerol, 100 mM dithiothreitol (DTT)). Proteins were resolved on 10% SDS-PAGE and then transferred onto polyvinylidene fluoride (PVDF) membranes. The PVDF membranes were blocked with 5% nonfat milk, followed by incubation with primary and secondary antibodies. The following antibodies were used: anti-NFIC (Cell Signaling Technology, Danvers, MA, USA, 11911S, 1:1000), anti-H3 (Cell Signaling Technology, 4499S, 1:1000), and anti-rabbit IgG (Bioker Biotechnology, Hangzhou, China, 2524, 1:5000).

### 2.8. Statistical Analysis

Expression of hub genes between the two groups was performed by unpaired t-tests using GraphPad Prism (version 8.3.0, GraphPad Software). *p* value < 0.05 was assigned as significance.

## 3. Results

### 3.1. Data Preprocessing and DEGs Screening

**Figure 2:**
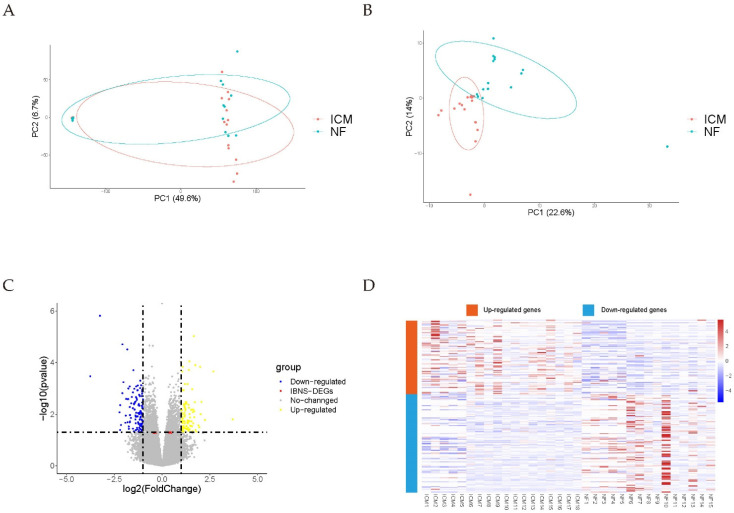
Merging of datasets and DEGs with the cutoff (*p* value < 0.05 and |(log_2_FC)| ≥ 1). (**A**,**B**) PCA plots before and after removal of the inter-batch effect. (**C**) Volcano plot. (**D**) Heatmap of all DEGs. NF, non-failing heart tissue; ICM, heart tissue of ischemic cardiomyopathy patient; DEGs, differentially expressed genes; IBNS-DEGs, important-but-not-such-significantly-differentially expressed genes.

Two RNA-seq datasets (GSE120852 [28], GSE55296 [29]), including 18 ICM and 15 NF samples, were combined with batch effects eliminated. In total, 208 differentially expressed genes (DEGs), including 118 downregulated and 90 upregulated genes, were identified in a meta-analysis using the strict cutoff (*p* value < 0.05, |(log_2_FC)| ≥ 1) (Table S4). Next, the batch effects were eliminated, the distributions of sample row counts before adjustment for batch effects and the distributions of DEGs after adjustment are illustrated in the PCA plots (Figure 2A,B). A volcano plot and DEGs heatmaps are shown in Figure 2C,D. The top 50 DEGs ordered by *p* value are presented in Table 1. Due to the variation in the human samples, we also used the loose cutoff (*p* value < 0.05, |(log_2_FC)| ≥ 0.7) to screen DEGs, including 201 upregulated and 255 downregulated genes (Table S5).

### 3.2. GO and KEGG Analyses of Downregulated Genes

**Figure 3:**
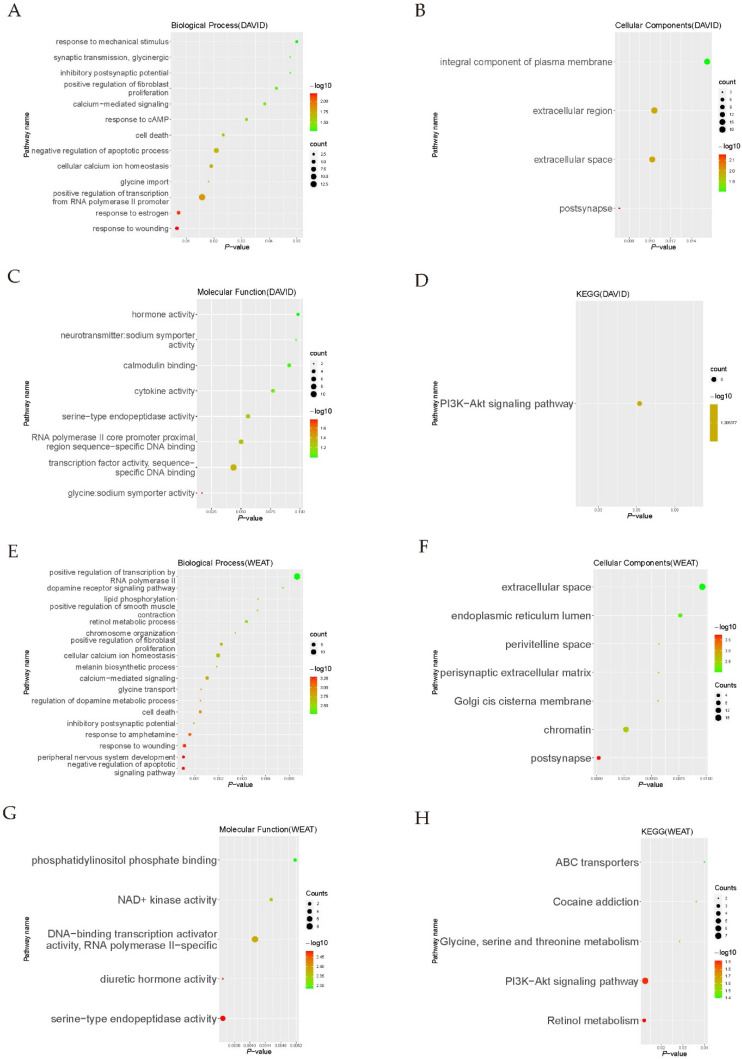
GO and KEGG analyses of downregulated genes screened by the strict cutoff. (**A**–**C**) DAVID GO analyses of downregulated genes. (**D**) DAVID KEGG analysis of downregulated genes. (**E**–**G**) WEAT GO analyses of downregulated genes. (**H**) WEAT KEGG analysis of downregulated genes. BP, biological process; CC, cellular component; MF, molecular function.

GO and KEGG analyses were performed by DAVID [30] for both upregulated and downregulated genes screened by the strict cutoff. The results of DAVID GO and KEGG analyses of the upregulated genes are shown in Figure S2. The upregulated genes were involved in the inflammatory response, extracellular region, transmembrane signaling receptor activity, antigen processing, and presentation pathways. More genes are downregulated in all DEGs. Additionally, we are more interested in downregulated transcription activators in which downstream genes would be also downregulated. In the DAVID GO analyses of all 118 downregulated genes, the most enriched biological process (BP) terms were associated with response to wounding, positive regulation of smooth muscle contraction, and positive regulation of transcription from RNA polymerase II promoter. The most enriched terms for cellular components (CC) were mainly associated with post synapse and extracellular space. The most enriched molecular function (MF) terms were associated with glycine: sodium symporter activity, transcription factor activity, sequence-specific DNA binding and RNA polymerase II core promoter proximal region sequence-specific DNA binding (Figure 3A–C). In the DAVID KEGG analysis, the downregulated genes were enriched in the PI3K-Akt signaling pathway (Figure 3D).

The DAVID GO and KEGG analysis for pathway enrichment analyses totally depend on the category count numbers of the gene list and one gene set, ignoring the different importance of every single gene. The weighted enrichment analysis (WEAT) method can overcome the issue of treating every gene equally to the conventional method [25]. The results of WEAT GO and KEGG analyses of the upregulated genes screened by the strict cutoff are shown in Figure S2. In the WEAT GO analyses of downregulated genes screened by the strict cutoff, the most enriched biological process (BP) terms were associated with negative regulation of the apoptotic signaling pathway, peripheral nervous system development, and response to wounding. The most enriched terms for cellular components (CC) were mainly associated with post synapse and chromatin. The most enriched molecular function (MF) terms were associated with serine-type endopeptidase activity, diuretic hormone activity and DNA-binding transcription activator activity, RNA polymerase II-specific. (Figure 3E–G). In the WEAT KEGG analysis, the downregulated genes screened by the strict cutoff were enriched in Retinol metabolism, ABC transporters, and PI3K-Akt signaling pathway (Figure 3H).

DAVID GO and KEGG analyses were also performed on DEGs screened by loose cutoff. The upregulated genes were enriched in inflammatory response, extracellular region, antigen processing, and presentation pathways, whereas the downregulated genes were involved in pathways such as positive regulation of smooth muscle contraction, extracellular space, transcription factor activity, sequence-specific DNA binding, and the PI3K-Akt signaling pathway (Figure S1). The enrichment results of DEGs screened by loose cutoff are similar to the DEGs screened by strict cutoff. Hence, we used the DEGs screened by the strict cutoff to undertake the following analysis.

### 3.3. Enrichment Analysis of NFIC-Binding Motif on Downregulated Promoter Sequences

In order to find out which transcription factor regulates the downregulated genes in ICM, previously obtained 118 downregulated genes from full DEGs were used to identify motifs significantly enriched in the promoter regions (−5000 bp relative to the TSS) via Simple Enrichment Analysis (SEA). Due to the limitation of the “biomaRt” package, some promoter regions could not be found and some regions were duplicated when extracting the promoter regions with it. Finally, 107 effective promoter regions were obtained. We acquired 220 motifs with *p* value < 0.005 through SEA. However, the expression of transcription factors with significantly enriched motifs may not decrease. In order to select downregulated important transcription factors, we set a loose cutoff as log_2_FC < −0.3 and *p* value < 0.06 to include more downregulated genes. Then we obtained four overlapping transcription factors (KLF6, KLF1, KLF7, and NFIC) with both enrichment in the downregulated gene promoters and decreased expression, shown in Table 2. However, these four transcription factors were not included in the DEGs list. A recent study pointed out that important-but-not-such-significantly-differentially expressed genes (IBNS-DEGs) may play important roles in the given biological process although it is not taken as candidate genes which can be recognized by Gene Importance Calculator (GIC) [31]. Thus, we calculated these four genes’ GIC scores, shown in Table 2. Interestingly, among the four transcription factors, NFIC was the most important one with the highest GIC score (Table 2). Summarizing the results of SEA and GIC analysis, NFIC would be an important upstream regulator of downregulated genes in ICM.

### 3.4. Verification of Predicted Genes with NFIC-Binding Motif by Validating Microarray Dataset and Nfic Knockdown Cardiomyocytes

**Figure 4:**
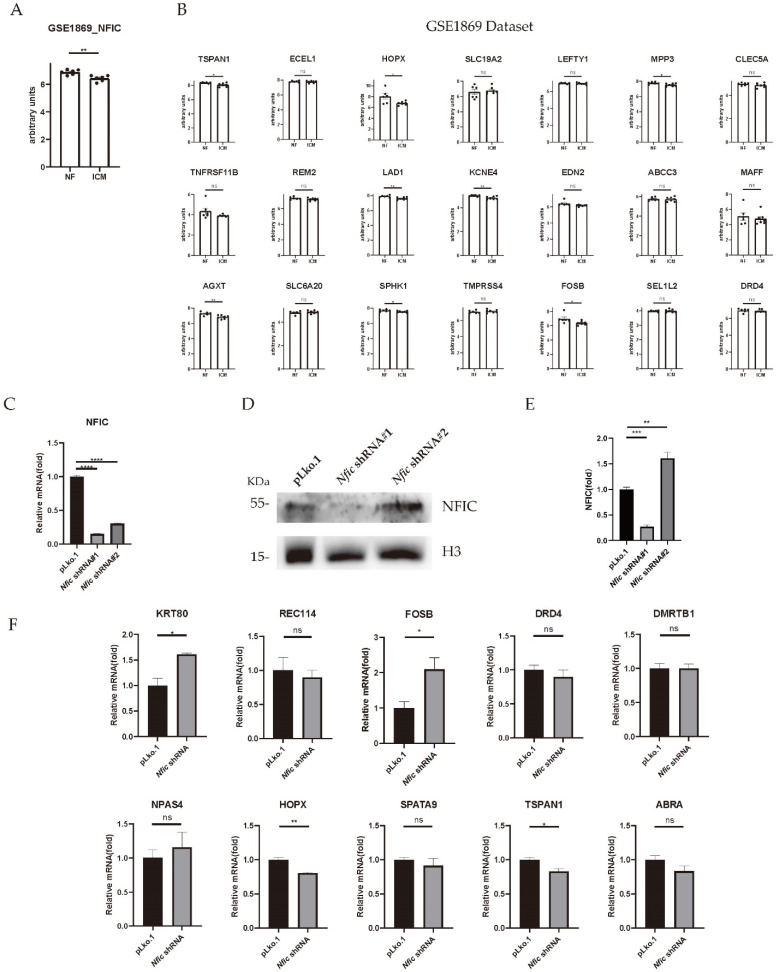
Verification of predicted genes with NFIC-binding motif. (**A**) The expression of NFIC in the validating GSE1869 dataset. (**B**) Expression of predicted genes with an NFIC-binding motif in the validating GSE1869 dataset. (**C**) The mRNA expression level of *Nfic* in H9C2 cells. (**D**) Western blot analysis of NFIC in H9C2 cells. (**E**) Quantification of the expression of NFIC in H9C2 cells. (**F**) The mRNA expression level of predicted genes in *Nfic* knockdown H9C2 cells (*n* = 3 per group). * *p* value < 0.05; ** *p* value < 0.01; *** *p* value < 0.001; **** *p* value < 0.0001; NF, normal heart tissue; ICM, heart tissue of ischemic cardiomyopathy patient; ns: no significance.

Among the 118 downregulated genes, 33 of them were predicted to have a NFIC-binding motif in SEA motif analysis (Table 3). In the validating dataset GSE1869, the expression of *NFIC* was significantly downregulated in ICM samples, and expressions of 21 predicted downstream genes were found whereas the other 12 genes were not recorded. Among the 21 genes, *TSPAN1*, *HOPX*, *MPP3*, *LAD1*, *KCNE4*, *AGXT*, *SPHK1,* and *FOSB* were significantly downregulated (Figure 4A,B).

In parallel, we performed cellular experiments to cross-validate our predication. We decreased the *Nfic* expression by lentivirus-mediated transgene expression of two *Nfic*-specific shRNA (shRNA#1 and shRNA#2) in cultured H9C2. qRT-PCR was performed to measure the expression levels of *Nfic* mRNA. Accordingly, mRNA expression level of *Nfic* was lower in H9C2 treated with *Nfic* shRNA than H9C2 treated with empty pLKO.1 vector. The knockdown efficiency was better using *Nfic* shRNA#1 demonstrated by the mRNA and protein expression levels (Figure 4C–E). Thus, the mRNA expression levels of all 33 predicted genes with NFIC-binding motif were measured in H9C2 treated with *Nfic* shRNA#1. Among them, two genes, *Tspan1* (tetraspanin1) and *Hopx* (HOP homeobox) were significantly downregulated (Figure 4F), whereas expression levels of others were not consistent with prediction or too low to be detected (Figure S3 and Table S8).

## 4. Discussion

In this study, we performed an integrated bioinformatics analysis of clinical ICM RNA-seq data. A total of 208 DEGs including 118 downregulated and 90 upregulated genes were identified by RNA-seq meta-analysis. Simple Enrichment Analysis and GIC were performed to identify one key transcription factor *NFIC*. Downstream genes were verified by quantitative real-time PCR experiments.

Among the top 50 significant DEGs (Table 1), several genes, such as *ERBB3*, *RCAN1*, and *NLRX1* were previously reported to be related with ICM [32,33,34,35,36]. *ERRB3*, erb-b2 receptor tyrosine kinase 3, has been reported as a protective factor which inhibits death mechanisms activated by redox stress and supports an involvement of this receptor in the pro-survival responses after MI/R injury [32]. The activation of NRG-1/ErbB3 signaling significantly decreased inflammation and improved LV function in remote ischemic conditioning rats [33]. Moreover, cardiomyocytes depleted of *RCAN1* were more sensitive to simulated MI/R and the calcineurin/Rcan1-signaling cascade could act as a potential therapeutic target through which to benefit from innate circadian changes in cardiac protection without disrupting core circadian oscillations that are essential to cardiovascular, metabolic, and mental health [34]. *NLRX1*, NOD-like receptors family member X1, is significantly downregulated following intestinal MI/R injury [35], and ablation of the mitochondrial *NLRX1* exerts a detrimental effect on acute cardiac infarction induced by a prolonged ischemia-reperfusion episode and activates potential MI/R injury mechanisms contributing to increased MI/R injury, related to elevated energy metabolism and diminished Akt [36].

Due to different criteria regarding experimental conditions, sample selection between different datasets, the gene expression levels always show diversity in different datasets. RNA-seq meta-analysis overcomes the above limitations well. In the merged datasets, we identified 208 overlapped DEGs. We are more interested in the downregulated genes and the upregulated genes remain to be studied. GO and KEGG analyses were performed by DAVID and WEAT. In general, the genes in one input gene list and one gene set are equally treated in gene functional enrichment analysis which would lead to biased results because genes are actually different in essentiality. WEAT provides a weighted gene functional enrichment analysis method according to the above truth that different genes have different essentiality in one biological process or an organ [25]. In WEAT analysis, we added different weights to our DEGs according to their essentiality in the heart. In the results of DAVID and WEAT BP analyses, there are same terms such as response to wounding, cell death, and positive regulation of transcription from the RNA polymerase II promoter, indicating that these processes have played an important role in the development of ICM. Terms such as positive regulation of smooth muscle contraction and lipid phosphorylation are only enriched in WEAT BP analysis. Additionally, it is reasonable that the movement and metabolism of muscle is damaged during the process of ICM. Thus, WEAT gives expression to the importance of essentiality scores in gene functional analysis. The PI3K-Akt signaling pathway is enriched in both DAVID and WEAT KEGG analyses. Previous studies have shown that the activation of the P13K/Akt signaling pathway enhances the cardioprotective effect of progranulin in the rat model of acute MI/R injury [37]. In our study, several genes (*DDIT4*, *MYC*, *CSF3*, *TNC*, *EIF4E1B*, *PCK2*, and *BDNF*) belonging to the P13K/Akt signaling pathway were downregulated in ICM, indicating the potential diagnostic and therapeutic targets of ICM. The WEAT KEGG analysis listed other pathways such as “Retinol metabolism” and “Glycine, serine and threonine metabolism” which predicted the latent metabolic characteristics of ICM.

Many studies have reported genes related to ICM. For example, *F13A1* and its protein product factor XIII-A (FXIII-A) is a member of the transglutaminase family of enzymes, named for its important role in blood coagulation [38]. The genetically determined FXIII-A level acts as an independent predictor of early prognosis after acute myocardial infarction (AMI) and the mutant of FXIII-A seems associated with lower AMI risk, which makes it a marker gene of AMI [39,40]. Moreover, it has a direct proangiogenic effect on endothelial cells in vitro due to the possible role of FXIII in inhibiting antiangiogenic TSP1 gene expression [41,42]. Additionally, modulation of FXIII activity by therapy influences myocardial healing in the mouse model of coronary ligation [42]. Few studies point out the regulators which regulate the ICM-related genes. Dysregulated transcription factors and their downstream gene expression signatures indicate the molecular mechanisms underlying disease processes. Therefore, finding such novel regulators aids in the development of new diagnostic and therapeutic applications. Transcription activators are cellular regulators that can upregulate the transcription level of genes by binding to specific short sequences of DNA in the promoters or enhancers of target genes. Many studies have reported that downregulated transcription activators play important roles in various diseases. Reduced expression of transcription factor *FOXP1* and its downstream gene p21^Waf1/Cip1^ was found to be a contributing factor in Huntington’s disease [43]. The expression of transcription activator FOXO4 is decreased significantly in most gastric cancer tissues and in various human gastric cancer cell lines, suggesting that it may serve as a potential therapeutic target for gastric cancer [44]. By the inspiration of these studies, we hope to find the key downregulated transcription activators and their downstream targets of ICM. Hence, we are more interested in the downregulated genes and transcription activators. NFIC was regarded as a key regulator of tooth development initially. It is expressed in odontoblasts of crown and root and the deletion of *Nfic* leads to short tooth roots [45]. However, the investigation of NFIC in the heart is still very limited. Liu Q et al. found that the addition of 13-cis-retinoic acid during cardiomyocyte differentiation would increase the binding of NFIC to DNA when studying the toxic mechanism of 13-cis-retinoic-acid-induced early cardiac differentiation and development [46]. Another member of the NFI family, NFIX, has been reported as a transcriptional switch from embryonic to fetal myogenesis [47] and directly represses the Myostatin promoter; thus, controlling the proper timing of satellite cell differentiation and muscle regeneration [48]. Similar to NFIX, NFIC binds and regulates downstream genes through the binding motif. It is likely that NFIC will bind and regulate the genes which have the NFIC binding motif in their promoter regions. In our study, we found that NFIC is highly expressed in the heart, but the role of NFIC in the heart is unclear. NFIC is a transcription factor which positively regulates the expression of downstream genes. In general, disease-related transcription factors are usually found in DEGs. However, the cutoff of DEGs is a little strict which may leave out some important genes. A recent study pointed out that important-but-not-such-significantly-differentially expressed genes (IBNS-DEGs) may play important roles in the given biological process although it is not taken as candidate genes which can be recognized by the Gene Importance Calculator [31]. Therefore, we adjusted the cutoff to search the key regulator. Our study performed the motif research combining Simple Enrichment Analysis and the Gene Importance Calculator. SEA showed that the NFIC-binding motif is significantly enriched in the promoter region of downregulated genes in ICM and GIC determined the high importance of NFIC. In summary, we proved that NFIC is an upstream regulator of downregulated genes in clinical ICM and plays an important role in many physiological and pathological processes in the heart.

The Kruppel-like Factor (KLF) family is a subfamily of the zinc-finger class of DNA-binding transcriptional regulators. There are several published reports describing the role of KLFs in the cardiovascular system. KLF6 was reported to cooperate with the related GC box binding protein Sp1 to regulate the expression of endoglin and related members of the TGF-β signaling complex in vascular repair [49]. KLF15 is an inhibitor of cardiac hypertrophy in which overexpression in neonatal rat ventricular cardiomyocytes inhibits cell size, protein synthesis, and hypertrophy-related gene expression [50]. Our study indicated that KLFs are important transcription factors and may regulate some momentous genes in ICM, but their function in ICM needs further study.

Many genes with the NFIC-binding motif were found through motif enrichment research but only two genes were verified, which showed the importance of these two genes. TSPAN1 (tetraspanin 1), a member of the tetraspanin (TSPAN/TM4SF) superfamily, was reported to be involved in many fundamental biological processes, such as cell proliferation, adhesion, and migration [51]. However, the role of TSPAN1 in the heart is unclear yet. Our study found that TSPAN1 is downregulated in ICM owing to the key transcription factor NFIC, indicating that it may be involved in the proliferation of cardiomyocytes and the remolding of myocardium in ICM. HOPX is a homeodomain protein which lacks residues for binding DNA. It was shown to be necessary for normal myocardial development in mice and zebrafish through studies with loss-of-function mutations [52]. Additionally, it contacts DNA indirectly by serum response factor or other DNA-binding proteins and represses genes through recruiting histone deacetylases [53].

There are some limitations in our study: (1) the accuracy of disease assessment and prediction can be improved if the sample size is increased and genetic information is completed; (2) verification in our study is not enough. For example, the luciferase reporter assay, ChIP assay, and in vivo animal experiments were in need of further study to verify the relationship between *NFIC* and ICM.

## 5. Conclusions

In summary, by integrating and analyzing different datasets, and through the cell experiment validation, one key regulator *NFIC* and its two downstream genes (*TSPAN1* and *HOPX*) of ICM, were identified. Through GO and KEGG analyses, the response to wounding, positive regulation of smooth muscle contraction, chromatin, PI3K-Akt signaling pathway, and transporters pathways may be related to ICM. Together, our findings provide new perspectives for the understanding of *NFIC* and its downstream genes in the pathogenesis of ICM.

## Figures and Tables

**Table 1:** Top 50 DEGs identified in the meta-analysis.

Ensembl_ID	Gene_Symbol	*p* Value	Average_Log_2_FC	Effect ^1^
ENSG00000125740	*FOSB*	0.0000015	−3.257547079	Down
ENSG00000175161	*CADM2*	0.0000197	−2.071624331	Down
ENSG00000176390	*CRLF3*	0.0000218	−0.658728667	Down
ENSG00000212901	*KRTAP3-1*	0.0000095	1.661253363	Up
ENSG00000256223	*ZNF10*	0.0000224	−0.484320108	Down
ENSG00000185022	*MAFF*	0.0000308	−1.821593264	Down
ENSG00000039537	*C6*	0.0001451	1.089126636	Up
ENSG00000103037	*SETD6*	0.0001428	−0.534750906	Down
ENSG00000147257	*GPC3*	0.0001270	1.743008369	Up
ENSG00000159200	*RCAN1*	0.0001080	−1.002579722	Down
ENSG00000161905	*ALOX15*	0.0000903	1.430983226	Up
ENSG00000176194	*CIDEA*	0.0001473	1.999963173	Up
ENSG00000197446	*CYP2F1*	0.0000935	0.979810226	Up
ENSG00000205085	*GARIN1A*	0.0001299	−0.984622408	Down
ENSG00000086289	*EPDR1*	0.0001936	1.404957629	Up
ENSG00000198570	*RD3*	0.0001984	−1.503858047	Down
ENSG00000062282	*DGAT2*	0.0002235	1.055794141	Up
ENSG00000244682	*FCGR2C*	0.0002228	2.694995426	Up
ENSG00000197006	*METTL9*	0.0002619	0.827325535	Up
ENSG00000006652	*IFRD1*	0.0003909	−0.881609917	Down
ENSG00000100167	*SEPTIN3*	0.0003299	−0.69613844	Down
ENSG00000108654	*DDX5*	0.0003928	−0.422221574	Down
ENSG00000160703	*NLRX1*	0.0003473	0.526989002	Up
ENSG00000162892	*IL24*	0.0003406	−3.76275497	Down
ENSG00000183648	*NDUFB1*	0.0003846	−0.834568054	Down
ENSG00000065361	*ERBB3*	0.0004149	−0.734170375	Down
ENSG00000123243	*ITIH5*	0.0004356	0.866063518	Up
ENSG00000026950	*BTN3A1*	0.0005153	0.841120801	Up
ENSG00000048162	*NOP16*	0.0005381	−0.967347096	Down
ENSG00000103404	*USP31*	0.0006926	−0.587270031	Down
ENSG00000109846	*CRYAB*	0.0006931	−0.858475283	Down
ENSG00000112164	*GLP1R*	0.0006748	1.339457951	Up
ENSG00000114062	*UBE3A*	0.0005701	−0.504043138	Down
ENSG00000127527	*EPS15L1*	0.0007000	0.412614122	Up
ENSG00000137033	*IL33*	0.0006977	0.738889805	Up
ENSG00000137338	*PGBD1*	0.0006317	−0.641698806	Down
ENSG00000153234	*NR4A2*	0.0005670	−2.024169309	Down
ENSG00000155893	*PXYLP1*	0.0004983	−1.144178122	Down
ENSG00000163444	*TMEM183A*	0.0006250	−0.468754302	Down
ENSG00000170242	*USP47*	0.0006670	−0.643634597	Down
ENSG00000184566	*NONE-*	0.0006732	1.200758974	Up
ENSG00000196757	*ZNF700*	0.0007092	−0.676810871	Down
ENSG00000142178	*SIK1*	0.0007473	−1.098038824	Down
ENSG00000258311	*NONE*	0.0007591	−1.27119987	Down
ENSG00000105221	*AKT2*	0.0008704	0.344111036	Up
ENSG00000113739	*STC2*	0.0008295	−1.35464027	Down
ENSG00000116761	*CTH*	0.0008316	−1.69781611	Down
ENSG00000128272	*ATF4*	0.0007886	−0.998749847	Down
ENSG00000144655	*CSRNP1*	0.0008495	−0.983291446	Down
ENSG00000167920	*KRT10-AS1*	0.0008554	0.855157383	Up

^1^: “Up” or “Down” indicates whether the gene was upregulated or downregulated.

**Table 2:** Binding motif and GIC analysis of four transcription factors.

Logo	Alt ID ^1^	*p* Value ^2^	TP ^3^	FP ^4^	Enrichment Ratio ^5^	Score Threshold ^6^	Average_Log_2_FC in RNA-seq	*p* Value in RNA-seq	GIC Score ^7^
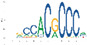	MA1517.1.KLF6	3.19 × 10^−7^	59/107 (55.14%)	23/107 (21.5%)	2.5	13	−0.83	0.0091	0.547
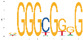	MA1870.1.KLF7	8.01 × 10^−4^	66/107 (61.68%)	42/107 (39.25%)	1.56	12	−0.54	0.038	0.746
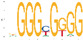	MA0493.2.KLF1	1.06 × 10^−4^	102/107 (95.33%)	83/107 (77.57%)	1.23	10	−0.81	0.026	0.541
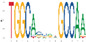	MA01119.1. NFIC::TLX1	3.39 × 10^−4^	33/107 (30.8%)	12/107 (11.2%)	2.62	9.31	−0.30	0.058	0.79

^1^: an alternate name for the motif that may be provided in the motif database file; ^2^: the optimal enrichment *p* value of the motif according to the statistical test; ^3^: the number of primary sequences matching the motif/the number of primary sequences (the percentage of primary sequences matching the motif); ^4^: the number of control sequences matching the motif/the number of control sequences (the percentage of control sequences matching the motif); ^5^: the relative enrichment ratio of the motif in the primary vs. control sequences; ^6^: a sequence is said to match the motif if some position within it has a match score greater than or equal to the optimal threshold (Score Threshold), this is the score threshold used by SEA to determine the values of “TP” and “FP”, SEA uses the hold-out sequence set to determine the score threshold unless there are too few sequences in the input. ^7^: represents the importance of the gene, higher GIC score represents more importance [31].

**Table 3:** Predicted genes with the NFIC-binding motif.

Gene Name	seq_Score ^1^	seq_Class ^2^
*SLC19A2*	17	tp
*LEFTY1*	14.18	tp
*BPIFB3*	13.5	tp
*ECEL1*	13.37	tp
*TRIM42*	13.11	tp
*KRT80*	11.84	tp
*REC114*	11.79	tp
*MPP3*	10.55	tp
*NXNL1*	10.2	tp
*ABCC12*	10.16	tp
*CLEC5A*	10.11	tp
*TNFRSF11B*	10.09	tp
*EIF4E1B*	10.05	tp
*REM2*	10.01	tp
*LAD1*	9.98	tp
*NPAS4*	9.93	tp
*KCNE4*	9.77	tp
*EDN2*	9.7	tp
*ABCC3*	9.6	tp
*MYRFL*	9.56	tp
*AGXT*	9.4	tp
*TSPAN1*	9.4	tp
*MAFF*	9.4	tp
*HOPX*	9.4	tp
*SPATA9*	9.4	tp
*DMRTB1*	9.4	tp
*SLC6A20*	9.4	tp
*SPHK1*	9.4	tp
*TMPRSS4*	9.4	tp
*FOSB*	9.4	tp
*SEL1L2*	9.4	tp
*DRD4*	9.4	tp
*ABRA*	9.4	tp

^1^: the seq_Score of a sequence is its maximum motif match score over all sequence positions. The motif match score of a position in a sequence is computed by summing the appropriate entry from each column of the position-dependent scoring matrix that represents the motif; ^2^: whether the sequence is a true positive, ‘tp’, or a false positive, ‘fp’.

## Data Availability

The data presented in this study are available upon request from the corresponding author.

## Supplementary Materials

The following supporting information can be downloaded at: https://www.mdpi.com/article/10.3390/genes13061051/s1, Figure S1: GO and KEGG analyses of DEGs with loose cutoff (*p* value < 0.05 and |(log_2_FC)| ≥ 0.7); Figure S2: GO and KEGG analyses of upregulated genes; Figure S3: The mRNA expression level of the rest of predicted genes which can be detected in *Nfic* knockdown H9C2 cells; Table S1: GSE120852 sample information; Table S2: GSE55296 sample information; Table S3: GSE1869 sample information; Table S4: The DEGs list screened by the strict cutoff (*p* value < 0.05 and |(log_2_FC)| ≥ 1); Table S5: The DEGs list screened by the loose cutoff (*p* value < 0.05 and |(log_2_FC)| ≥ 0.7); Table S6: Primer information; Table S7: *Nfic* shRNA information; Table S8: The cycle threshold (CT) values of qRT-PCR assay.

## Abbreviations

*NFIC*, Nuclear Factor I C; GO, gene ontology; KEGG, Kyoto Encyclopedia of Genes and Genomes; DEGs, differentially expressed genes; BP, biological process; CC, cellular component; MF, molecular function; PCA, principal component analysis; DAVID, Database for Annotation, Visualization and Integrated Discovery tool; WEAT, Weighted Enrichment Analysis Tools; SEA, Simple Enrichment Analysis; GIC, Gene Importance Calculator.
