# Supplementary material for: Bioinformatics and Experimental Analyses Reveal NFIC as an Upstream Transcriptional Regulator for Ischemic Cardiomyopathy
Source: Genes (Basel). 2022 Jun 13;13(6):1051. doi: 10.3390/genes13061051 (PMC9222441; doi:10.3390/genes13061051)
Supplement: Supplementary file 1 [file genes-13-01051-s001.zip › genes-1680104-supplementary/Supplementary Figure .pdf]

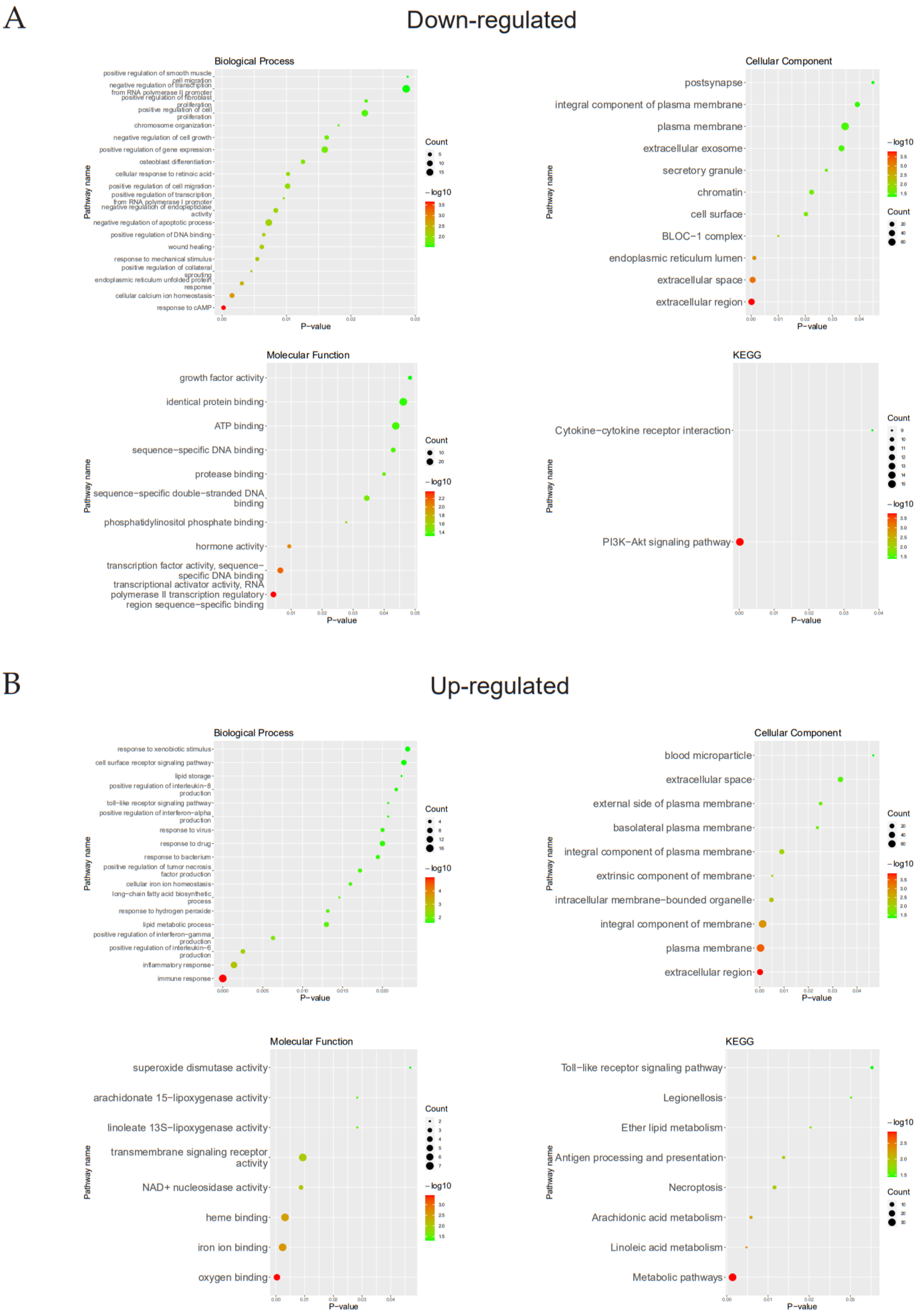

Figure S1. GO and KEGG analyses of DEGs with loose cutoff（p value < 0.05 and ︱(log2 FC) ︱ ≥ 0.7）. (A) GO and KEGG analyses of down-regulated genes. (B) GO and KEGG analyses of up-regulated genes

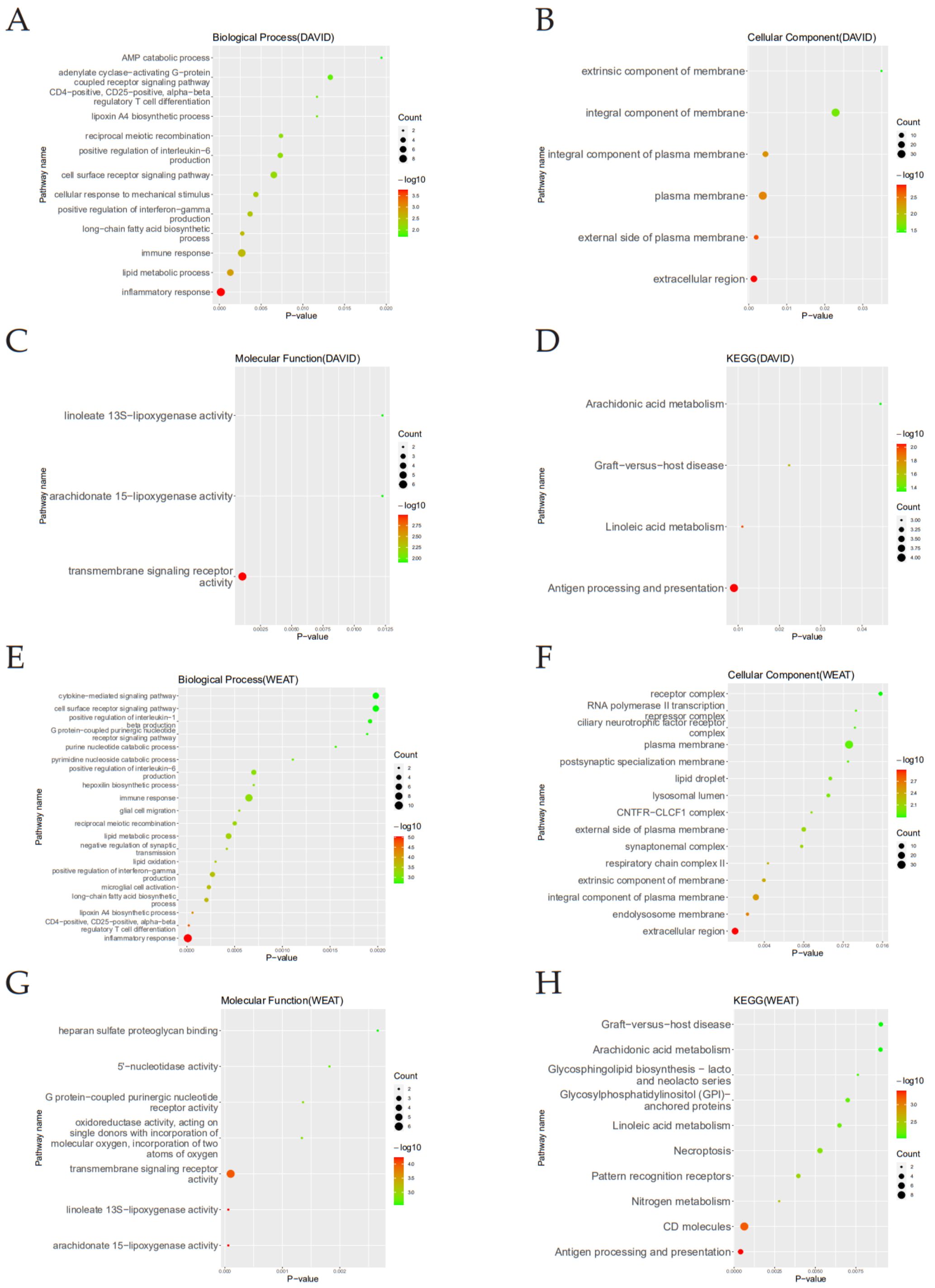

Figure S2. GO and KEGG analyses of up-regulated genes. (A-C) DAVID GO analyses of up-regulated genes. (D) DAVID KEGG analysis of up-regulated genes. (E-G) WEAT GO analyses of up-regulated genes. (H) WEAT KEGG analysis of up-regulated genes.

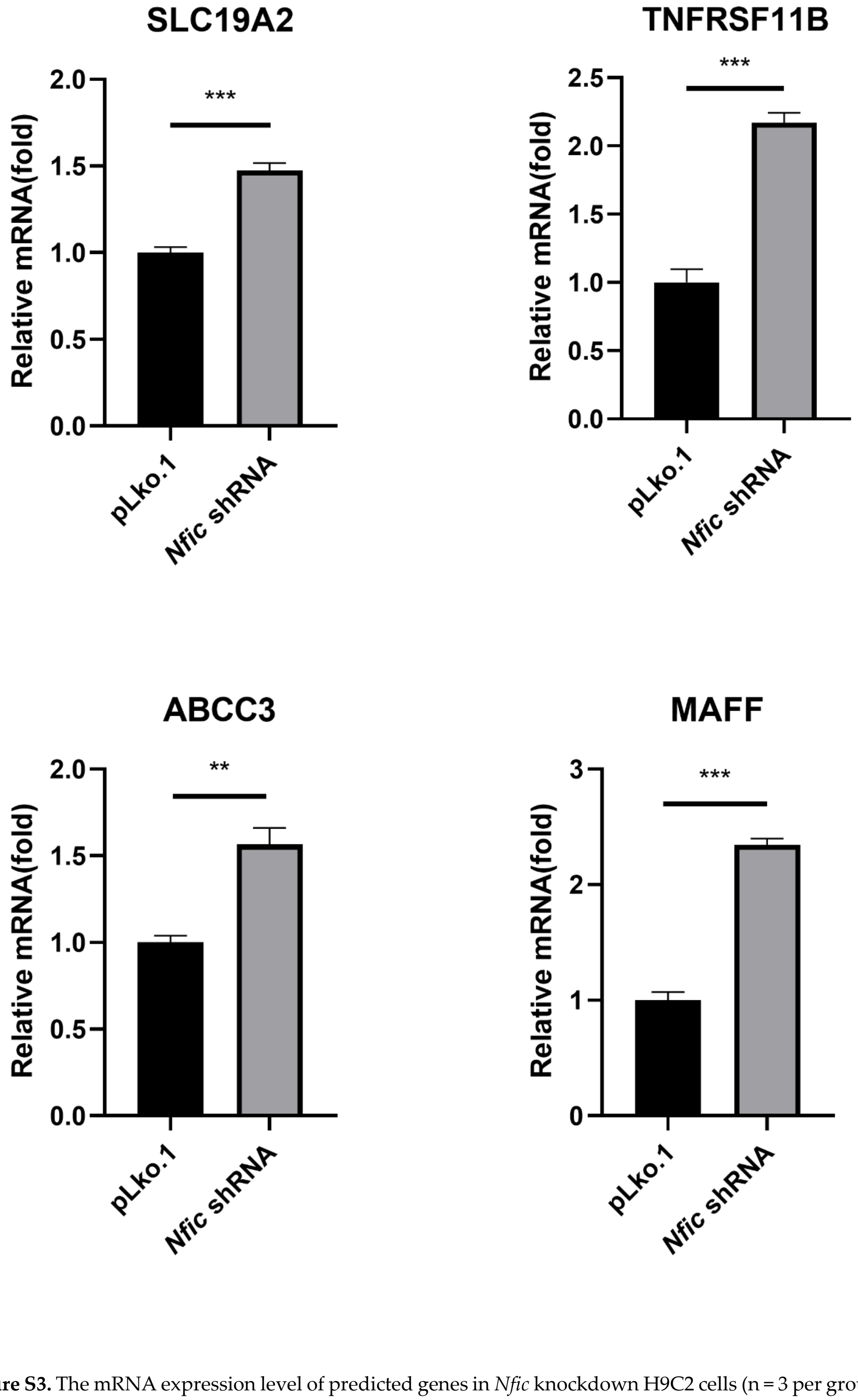

Figure S3. The mRNA expression level of predicted genes in *Nfic* knockdown H9C2 cells (n = 3 per group)
