# Supplementary material for: Bioinformatics and Experimental Analyses Reveal NFIC as an Upstream Transcriptional Regulator for Ischemic Cardiomyopathy
Source: Genes (Basel). 2022 Jun 13;13(6):1051. doi: 10.3390/genes13061051 (PMC9222441; doi:10.3390/genes13061051)
Supplement: Supplementary file 1 [file genes-13-01051-s001.zip › genes-1680104-supplementary/Supplementary Table.pdf]

Table S1 GSE120852 sample information

Group	GEO Accession	Organism	Platform ID	Disease state	Tissue
NF_LV1	GSM3417081	Homo sapiens	GPL16791	normal control	heart
NF_LV2	GSM3417082	Homo sapiens	GPL16791	normal control	heart
NF_LV3	GSM3417083	Homo sapiens	GPL16791	normal control	heart
NF_LV4	GSM3417084	Homo sapiens	GPL16791	normal control	heart
NF_LV5	GSM3417085	Homo sapiens	GPL16791	normal control	heart
LV-HF_LV1	GSM3417086	Homo sapiens	GPL16791	end-stage ischemic cardiomyopathy	heart
LV-HF_LV2	GSM3417087	Homo sapiens	GPL16791	end-stage ischemic cardiomyopathy	heart
LV-HF_LV3	GSM3417088	Homo sapiens	GPL16791	end-stage ischemic cardiomyopathy	heart
LV-HF_LV4	GSM3417089	Homo sapiens	GPL16791	end-stage ischemic cardiomyopathy	heart
LV-HF_LV5	GSM3417090	Homo sapiens	GPL16791	end-stage ischemic cardiomyopathy	heart

Table S2 GSE55296 sample information

Group	GEO Accession	Organism	Platform ID	Disease state	Tissue
Ischemic rep1	GSM1333746	Homo sapiens	GPL16288	ischemic	heart
Ischemic rep2	GSM1333748	Homo sapiens	GPL16288	ischemic	heart
Ischemic rep3	GSM1333750	Homo sapiens	GPL16288	ischemic	heart
Ischemic rep4	GSM1333755	Homo sapiens	GPL16288	ischemic	heart
Ischemic rep5	GSM1333758	Homo sapiens	GPL16288	ischemic	heart
Ischemic rep6	GSM1333759	Homo sapiens	GPL16288	ischemic	heart
Ischemic rep7	GSM1333760	Homo sapiens	GPL16288	ischemic	heart
Ischemic rep8	GSM1333761	Homo sapiens	GPL16288	ischemic	heart
Ischemic rep9	GSM1333763	Homo sapiens	GPL16288	ischemic	heart
Ischemic rep10	GSM1333764	Homo sapiens	GPL16288	ischemic	heart
Ischemic rep11	GSM1333766	Homo sapiens	GPL16288	ischemic	heart
Ischemic rep12	GSM1333769	Homo sapiens	GPL16288	ischemic	heart
Ischemic rep13	GSM1333775	Homo sapiens	GPL16288	ischemic	heart
Control rep1	GSM1333749	Homo sapiens	GPL16288	normal control	heart
Control rep2	GSM1333751	Homo sapiens	GPL16288	normal control	heart
Control rep3	GSM1333752	Homo sapiens	GPL16288	normal control	heart
Control rep4	GSM1333753	Homo sapiens	GPL16288	normal control	heart
Control rep5	GSM1333754	Homo sapiens	GPL16288	normal control	heart
Control rep6	GSM1333757	Homo sapiens	GPL16288	normal control	heart
Control rep7	GSM1333762	Homo sapiens	GPL16288	normal control	heart
Control rep8	GSM1333770	Homo sapiens	GPL16288	normal control	heart
Control rep9	GSM1333771	Homo sapiens	GPL16288	normal control	heart
Control rep10	GSM1333774	Homo sapiens	GPL16288	normal control	heart

Table S3 GSE1869 sample information

Group	GEO Accession	Organism	Platform ID	Disease state	Tissue
N1	GSM33108	Homo sapiens	GPL96	normal control	heart
N22	GSM33109	Homo sapiens	GPL96	normal control	heart
N26	GSM33110	Homo sapiens	GPL96	normal control	heart
N27	GSM33111	Homo sapiens	GPL96	normal control	heart
N28	GSM33112	Homo sapiens	GPL96	normal control	heart
N29	GSM33113	Homo sapiens	GPL96	normal control	heart
T1013i	GSM33116	Homo sapiens	GPL96	ischemic cardiomyopathy	heart
T1015i	GSM33117	Homo sapiens	GPL96	ischemic cardiomyopathy	heart
T1017i	GSM33118	Homo sapiens	GPL96	ischemic cardiomyopathy	heart
TEx1i	GSM33119	Homo sapiens	GPL96	ischemic cardiomyopathy	heart
TEx9i	GSM33120	Homo sapiens	GPL96	ischemic cardiomyopathy	heart

TEx10i	GSM33121	Homo sapiens	GPL96	ischemic cardiomyopathy	heart
TEx15i	GSM33122	Homo sapiens	GPL96	ischemic cardiomyopathy	heart

Table S4 The DEGs list screened by the strict cutoff (p value < 0.05 and | (log2FC) | ≥ 1)

Ensembl gene ID	log2FC	p value
ENSG00000212901	1.661253363	9.48129E-06
ENSG00000039537	1.089126636	0.000145125
ENSG00000147257	1.743008369	0.000127034
ENSG00000161905	1.430983226	9.03449E-05
ENSG00000176194	1.999963173	0.000147315
ENSG00000086289	1.404957629	0.000193605
ENSG00000062282	1.055794141	0.000223474
ENSG00000244682	2.694995426	0.000222836
ENSG00000112164	1.339457951	0.000674804
ENSG00000184566	1.200758974	0.000673207
ENSG00000153162	1.015508231	0.001056646
ENSG00000204366	1.581595929	0.00105809
ENSG00000163218	1.094032133	0.001152182
ENSG00000164342	1.129500942	0.00115174
ENSG00000247121	2.063603443	0.003299009
ENSG00000122756	1.250479168	0.003347586
ENSG00000240403	1.71385305	0.003371425
ENSG00000132881	1.236867017	0.003881255
ENSG00000204116	1.277705118	0.003826414
ENSG00000111291	1.151459429	0.004047506
ENSG00000154080	1.390350001	0.004269164
ENSG00000171823	1.145312112	0.004393035
ENSG00000102466	1.183852174	0.005040681
ENSG00000168421	1.405219545	0.005550407
ENSG00000182185	1.199052819	0.005704814
ENSG00000100462	1.238567896	0.006403048
ENSG00000116981	1.208893561	0.007261497
ENSG00000122224	1.629745006	0.006585548
ENSG00000143452	1.523416532	0.00673213
ENSG00000168079	1.012075818	0.007039748
ENSG00000174946	1.687893174	0.007691371
ENSG00000228672	1.043681933	0.006808447
ENSG00000203709	1.61705551	0.008330651
ENSG00000112837	1.055173983	0.008972498
ENSG00000198734	1.16437923	0.008894326
ENSG00000162877	1.442209413	0.009763193
ENSG00000143252	1.018546617	0.010019614
ENSG00000170075	2.021516465	0.009925229
ENSG00000138400	1.071592717	0.010289782
ENSG00000167447	1.045388716	0.012716455
ENSG00000169860	1.196107369	0.010660189
ENSG00000244734	1.178443701	0.012541355
ENSG00000163751	1.464288467	0.013372974
ENSG00000174885	1.685479888	0.013001567
ENSG00000254737	1.056606522	0.014582819
ENSG00000007314	1.091135954	0.015014391
ENSG00000144820	3.705568505	0.015796145

ENSG00000172724	2.248072456	0.016674689
ENSG00000100206	1.101281627	0.018743129
ENSG00000109971	1.339433112	0.017217757
ENSG00000178222	1.420519452	0.01818709
ENSG00000181036	1.178921159	0.018418047
ENSG00000183778	1.54125037	0.018880219
ENSG00000215790	1.012823938	0.01779001
ENSG00000101916	1.398707926	0.01936682
ENSG00000135318	1.036252955	0.020642379
ENSG00000099290	1.069728019	0.021979983
ENSG00000114200	1.189086521	0.022378687
ENSG00000152315	1.348051935	0.022277255
ENSG00000156886	1.634715728	0.022731791
ENSG00000223802	1.324603222	0.020925532
ENSG00000186399	1.906029374	0.026942725
ENSG00000107159	1.468611178	0.027528249
ENSG00000108309	1.269123961	0.028821493
ENSG00000214106	1.28053323	0.028711736
ENSG00000261272	1.127114425	0.028835286
ENSG00000204147	1.562923935	0.029282378
ENSG00000229644	1.51295227	0.029410197
ENSG00000116983	1.071468648	0.030025925
ENSG00000172232	1.9493254	0.031999422
ENSG00000125498	1.357829293	0.033333574
ENSG00000160868	1.090270825	0.034018421
ENSG00000180574	1.155111933	0.034239564
ENSG00000182890	1.207463981	0.03480062
ENSG00000081985	1.442707242	0.039261702
ENSG00000117215	1.754995175	0.040677769
ENSG00000125538	1.285285	0.03698161
ENSG00000135903	1.441182307	0.038425626
ENSG00000136682	1.382767748	0.040198558
ENSG00000141665	1.08081984	0.037625579
ENSG00000147255	1.030539747	0.037793584
ENSG00000181333	1.284396971	0.037779205
ENSG00000125910	1.072676106	0.043364726
ENSG00000100298	1.382419604	0.043612324
ENSG00000141433	1.049066077	0.043736678
ENSG00000179593	1.131079493	0.045062603
ENSG00000180549	1.594887969	0.045123564
ENSG00000189013	1.800036381	0.046933811
ENSG00000235531	1.11745342	0.047298484
ENSG00000181781	1.517646508	0.049500503
ENSG00000125740	-3.257547079	1.53328E-06
ENSG00000175161	-2.071624331	1.97026E-05
ENSG00000185022	-1.821593264	3.08424E-05
ENSG00000159200	-1.002579722	0.000108023
ENSG00000198570	-1.503858047	0.000198441
ENSG00000162892	-3.76275497	0.000340558
ENSG00000153234	-2.024169309	0.000567011
ENSG00000155893	-1.144178122	0.000498286
ENSG00000142178	-1.098038824	0.000747345

ENSG00000258311	-1.27119987	0.000759129
ENSG00000113739	-1.35464027	0.000829454
ENSG00000116761	-1.69781611	0.000831563
ENSG00000117479	-1.146703408	0.000960259
ENSG00000170091	-1.763897389	0.00099697
ENSG00000186567	-1.453698983	0.001022831
ENSG00000100146	-1.297320385	0.001169049
ENSG00000152049	-1.045539995	0.001137492
ENSG00000158486	-1.275470432	0.001155092
ENSG00000077044	-1.044446562	0.001333326
ENSG00000176170	-1.523835434	0.001420175
ENSG00000187527	-2.079093929	0.001526648
ENSG00000116741	-1.049049282	0.00159331
ENSG00000075426	-1.059869953	0.001962178
ENSG00000117472	-1.200587474	0.001759478
ENSG00000176697	-1.163340741	0.001972611
ENSG00000129455	-1.582626606	0.002121407
ENSG00000258227	-1.367053342	0.002155825
ENSG00000080166	-1.821420839	0.002594732
ENSG00000106366	-1.880860706	0.002514378
ENSG00000157017	-1.000129846	0.002638015
ENSG00000171241	-1.329670105	0.002432021
ENSG00000181649	-1.770052873	0.002582505
ENSG00000143006	-1.382965216	0.003175756
ENSG00000173826	-1.31502267	0.002735158
ENSG00000174429	-1.409162925	0.003353529
ENSG00000140403	-1.051067585	0.003484327
ENSG00000108846	-1.617720454	0.003772917
ENSG00000140563	-1.470329502	0.003843803
ENSG00000142871	-1.442922839	0.003701421
ENSG00000108342	-2.19484782	0.004430251
ENSG00000149300	-1.467070943	0.004149242
ENSG00000161647	-1.028058669	0.004515229
ENSG00000167634	-1.21803393	0.004005208
ENSG00000172020	-1.237687757	0.004416029
ENSG00000083454	-1.191452097	0.004658286
ENSG00000140450	-1.063249286	0.004686897
ENSG00000106258	-1.024822595	0.005016107
ENSG00000164761	-1.695190715	0.005225797
ENSG00000164611	-1.52965133	0.00579318
ENSG00000184205	-1.026051288	0.005279386
ENSG00000085465	-1.030396218	0.005862897
ENSG00000021852	-1.441004351	0.006540671
ENSG00000179165	-1.129063625	0.007508659
ENSG00000180525	-1.106121824	0.007646921
ENSG00000244623	-2.229507314	0.008383172
ENSG00000188517	-1.177418287	0.008496731
ENSG00000168209	-1.358286363	0.008808175
ENSG00000175264	-1.073381636	0.008839215
ENSG00000184254	-1.156937446	0.00881479
ENSG00000213988	-1.254993975	0.008950735
ENSG00000147588	-1.017339135	0.009358436

ENSG00000100889	-1.089709958	0.009688149
ENSG00000248713	-1.195890107	0.00997692
ENSG00000041982	-1.321881962	0.011979727
ENSG00000134668	-1.275641162	0.012041177
ENSG00000141314	-1.114844333	0.012463211
ENSG00000159166	-1.031210062	0.01082126
ENSG00000170561	-1.340320044	0.012337429
ENSG00000171773	-1.381569806	0.012391597
ENSG00000174576	-1.680621817	0.010722651
ENSG00000175894	-1.39922638	0.01125084
ENSG00000189120	-1.903583817	0.011296842
ENSG00000118985	-1.141349485	0.013584045
ENSG00000171551	-1.632634521	0.01384253
ENSG00000171476	-1.065272664	0.015953705
ENSG00000166268	-1.397781189	0.01598527
ENSG00000139835	-1.16994372	0.01618297
ENSG00000136997	-1.053418698	0.016341381
ENSG00000102104	-1.048124771	0.01755383
ENSG00000148798	-1.684830876	0.018998283
ENSG00000155890	-1.013328187	0.018031359
ENSG00000140798	-1.257791395	0.020205936
ENSG00000127129	-1.695710725	0.020474433
ENSG00000243709	-1.811246386	0.02050464
ENSG00000100312	-1.382305861	0.02224815
ENSG00000101251	-1.922695005	0.021319821
ENSG00000175766	-1.095566361	0.023131879
ENSG00000178734	-1.022599277	0.022315556
ENSG00000197561	-1.251110872	0.021115385
ENSG00000186190	-1.240150898	0.02331987
ENSG00000183324	-1.222235377	0.023646518
ENSG00000135437	-1.153921057	0.023926038
ENSG00000196517	-1.063552659	0.025324796
ENSG00000173124	-2.152981471	0.026044127
ENSG00000266258	-1.16101567	0.028042671
ENSG00000069696	-1.095967198	0.028330438
ENSG00000184697	-1.743729616	0.02975076
ENSG00000160282	-1.029139684	0.030745557
ENSG00000183117	-1.034226547	0.031231588
ENSG00000163817	-1.014456717	0.032957216
ENSG00000181433	-1.314139431	0.033069079
ENSG00000050628	-1.197572581	0.033523116
ENSG00000107105	-1.373397852	0.041189784
ENSG00000112494	-2.200510123	0.040830539
ENSG00000120937	-1.73760142	0.036741678
ENSG00000125931	-1.127427191	0.037847507
ENSG00000143867	-1.025474691	0.040506564
ENSG00000167767	-1.522961424	0.036147245
ENSG00000172482	-1.075938525	0.040619787
ENSG00000221819	-1.30147832	0.038398443
ENSG00000222005	-1.031887021	0.041930677
ENSG00000157315	-1.211992919	0.04205383
ENSG00000179388	-1.035883521	0.043359073

ENSG00000171495	-1.475510824	0.045895281
ENSG00000216895	-1.072213373	0.046738299
ENSG00000145757	-1.241982642	0.048221699
ENSG00000139890	-1.152815745	0.04936772
ENSG00000137648	-1.119332615	0.049708653

Table S5 The DEGs list screened by the loose cutoff (p value < 0.05 and | (log2FC) | ≥ 0.7)

Ensembl gene ID	log2FC	p value
ENSG00000212901	1.661253363	9.48129E-06
ENSG00000039537	1.089126636	0.000145125
ENSG00000147257	1.743008369	0.000127034
ENSG00000161905	1.430983226	9.03449E-05
ENSG00000176194	1.999963173	0.000147315
ENSG00000197446	0.979810226	9.35355E-05
ENSG00000086289	1.404957629	0.000193605
ENSG00000062282	1.055794141	0.000223474
ENSG00000244682	2.694995426	0.000222836
ENSG00000197006	0.827325535	0.000261878
ENSG00000123243	0.866063518	0.000435612
ENSG00000026950	0.841120801	0.000515258
ENSG00000112164	1.339457951	0.000674804
ENSG00000137033	0.738889805	0.000697704
ENSG00000184566	1.200758974	0.000673207
ENSG00000167920	0.855157383	0.000855394
ENSG00000153162	1.015508231	0.001056646
ENSG00000204366	1.581595929	0.00105809
ENSG00000163218	1.094032133	0.001152182
ENSG00000164342	1.129500942	0.00115174
ENSG00000164946	0.834170718	0.001193404
ENSG00000100804	0.931048711	0.001883266
ENSG00000109610	0.720063501	0.00192706
ENSG00000115252	0.875412034	0.002022141
ENSG00000161955	0.945276473	0.002044207
ENSG00000172164	0.943122013	0.001890546
ENSG00000181191	0.888123569	0.00245624
ENSG00000116663	0.736494729	0.003021973
ENSG00000138449	0.897181798	0.003248234
ENSG00000158270	0.804002082	0.002998482
ENSG00000168778	0.736644872	0.003274612
ENSG00000247121	2.063603443	0.003299009
ENSG00000122756	1.250479168	0.003347586
ENSG00000240403	1.71385305	0.003371425
ENSG00000108733	0.905444707	0.003534478
ENSG00000132881	1.236867017	0.003881255
ENSG00000204116	1.277705118	0.003826414
ENSG00000111291	1.151459429	0.004047506
ENSG00000154080	1.390350001	0.004269164
ENSG00000164307	0.713329866	0.004094274
ENSG00000171823	1.145312112	0.004393035
ENSG00000242265	0.796439464	0.004510037
ENSG00000102466	1.183852174	0.005040681
ENSG00000131374	0.885930038	0.004921476

ENSG00000111674	0.784948048	0.005610868
ENSG00000168421	1.405219545	0.005550407
ENSG00000182185	1.199052819	0.005704814
ENSG00000183508	0.84579168	0.005409412
ENSG00000143552	0.80972438	0.006009363
ENSG00000100462	1.238567896	0.006403048
ENSG00000106780	0.890418193	0.006637863
ENSG00000114670	0.773869156	0.007329536
ENSG00000116981	1.208893561	0.007261497
ENSG00000122224	1.629745006	0.006585548
ENSG00000124212	0.879603282	0.007029967
ENSG00000139737	0.731953729	0.006640082
ENSG00000141338	0.718335032	0.007529475
ENSG00000143036	0.971518964	0.006800509
ENSG00000143452	1.523416532	0.00673213
ENSG00000168079	1.012075818	0.007039748
ENSG00000174946	1.687893174	0.007691371
ENSG00000180488	0.756636319	0.007134093
ENSG00000228672	1.043681933	0.006808447
ENSG00000007350	0.820946863	0.008008041
ENSG00000116194	0.999768331	0.008352032
ENSG00000203709	1.61705551	0.008330651
ENSG00000254772	0.899337153	0.008453839
ENSG00000112837	1.055173983	0.008972498
ENSG00000198734	1.16437923	0.008894326
ENSG00000163131	0.912299366	0.009147598
ENSG00000176788	0.819259876	0.009102316
ENSG00000112096	0.75706914	0.009586275
ENSG00000162877	1.442209413	0.009763193
ENSG00000143252	1.018546617	0.010019614
ENSG00000159625	0.933358312	0.010000608
ENSG00000170075	2.021516465	0.009925229
ENSG00000181804	0.779479476	0.010110043
ENSG00000138400	1.071592717	0.010289782
ENSG00000065320	0.850056774	0.010666209
ENSG00000115415	0.916110682	0.012121358
ENSG00000139547	0.904943282	0.011145931
ENSG00000140090	0.885163904	0.012049483
ENSG00000167447	1.045388716	0.012716455
ENSG00000169860	1.196107369	0.010660189
ENSG00000185164	0.832363208	0.012306382
ENSG00000197296	0.850261624	0.012381281
ENSG00000197646	0.841445461	0.01166694
ENSG00000244734	1.178443701	0.012541355
ENSG00000115758	0.858759768	0.013094245
ENSG00000137563	0.92908525	0.013156576
ENSG00000145569	0.878725476	0.0135869
ENSG00000163751	1.464288467	0.013372974
ENSG00000174885	1.685479888	0.013001567
ENSG00000176994	0.893603141	0.013697188
ENSG00000188064	0.762285462	0.013792555
ENSG00000205038	0.9437182	0.013991928

ENSG00000066926	0.766016862	0.014120249
ENSG00000125730	0.812189369	0.014408042
ENSG00000172264	0.917081079	0.014582687
ENSG00000254737	1.056606522	0.014582819
ENSG00000007314	1.091135954	0.015014391
ENSG00000106682	0.755446245	0.015090641
ENSG00000110436	0.775350948	0.015440024
ENSG00000174738	0.74015805	0.015500321
ENSG00000144820	3.705568505	0.015796145
ENSG00000148488	0.795195078	0.015933916
ENSG00000153446	0.772897297	0.016165557
ENSG00000166347	0.726466538	0.016097151
ENSG00000174125	0.931241759	0.016125322
ENSG00000146411	0.738124331	0.016252723
ENSG00000172724	2.248072456	0.016674689
ENSG00000100206	1.101281627	0.018743129
ENSG00000109971	1.339433112	0.017217757
ENSG00000115365	0.818633961	0.01693523
ENSG00000146858	0.821305145	0.017251652
ENSG00000149527	0.752238385	0.017051692
ENSG00000152582	0.923348945	0.017711557
ENSG00000169758	0.90678775	0.017074183
ENSG00000178222	1.420519452	0.01818709
ENSG00000181036	1.178921159	0.018418047
ENSG00000183778	1.54125037	0.018880219
ENSG00000198326	0.837181722	0.018768038
ENSG00000215790	1.012823938	0.01779001
ENSG00000091262	0.772042665	0.019347216
ENSG00000101916	1.398707926	0.01936682
ENSG00000158158	0.726653275	0.019238084
ENSG00000135318	1.036252955	0.020642379
ENSG00000082074	0.900046744	0.022058129
ENSG00000099290	1.069728019	0.021979983
ENSG00000114200	1.189086521	0.022378687
ENSG00000128536	0.77640015	0.022859978
ENSG00000136960	0.70094631	0.022911024
ENSG00000143126	0.782130843	0.021911192
ENSG00000152315	1.348051935	0.022277255
ENSG00000156886	1.634715728	0.022731791
ENSG00000188021	0.757903044	0.021996254
ENSG00000223802	1.324603222	0.020925532
ENSG00000162407	0.748327677	0.023574674
ENSG00000180448	0.713190811	0.023922538
ENSG00000152782	0.753238057	0.025235287
ENSG00000180481	0.844612952	0.025736294
ENSG00000185818	0.893270514	0.025527183
ENSG00000109743	0.722524844	0.026464714
ENSG00000186399	1.906029374	0.026942725
ENSG00000148204	0.733843565	0.027231768
ENSG00000107159	1.468611178	0.027528249
ENSG00000108309	1.269123961	0.028821493
ENSG00000138336	0.767791473	0.028937195

ENSG00000172840	0.719872266	0.029014412
ENSG00000214106	1.28053323	0.028711736
ENSG00000261272	1.127114425	0.028835286
ENSG00000152207	0.972619805	0.029238643
ENSG00000204147	1.562923935	0.029282378
ENSG00000229644	1.51295227	0.029410197
ENSG00000116983	1.071468648	0.030025925
ENSG00000131037	0.857204554	0.030037156
ENSG00000172232	1.9493254	0.031999422
ENSG00000129636	0.71184434	0.032104941
ENSG00000087495	0.837803941	0.032722493
ENSG00000156345	0.748344949	0.032833722
ENSG00000125498	1.357829293	0.033333574
ENSG00000146476	0.743255688	0.035325859
ENSG00000150471	0.761864583	0.035179605
ENSG00000160868	1.090270825	0.034018421
ENSG00000180574	1.155111933	0.034239564
ENSG00000182890	1.207463981	0.03480062
ENSG00000198088	0.818962178	0.035245139
ENSG00000213760	0.744865176	0.034810061
ENSG00000081985	1.442707242	0.039261702
ENSG00000107242	0.850487722	0.040942067
ENSG00000112234	0.701080148	0.036268341
ENSG00000112796	0.909315148	0.037489557
ENSG00000116711	0.816882436	0.037341828
ENSG00000117215	1.754995175	0.040677769
ENSG00000123600	0.702337302	0.036129552
ENSG00000125538	1.285285	0.03698161
ENSG00000135903	1.441182307	0.038425626
ENSG00000136682	1.382767748	0.040198558
ENSG00000137168	0.834793665	0.040678212
ENSG00000138663	0.756322291	0.036541676
ENSG00000141665	1.08081984	0.037625579
ENSG00000147255	1.030539747	0.037793584
ENSG00000167840	0.751580875	0.039611169
ENSG00000169891	0.726199171	0.039604821
ENSG00000181333	1.284396971	0.037779205
ENSG00000213462	0.955756376	0.041242312
ENSG00000153094	0.729309346	0.041897573
ENSG00000164532	0.857992476	0.042018418
ENSG00000206172	0.857580457	0.042170738
ENSG00000125910	1.072676106	0.043364726
ENSG00000100298	1.382419604	0.043612324
ENSG00000141433	1.049066077	0.043736678
ENSG00000179593	1.131079493	0.045062603
ENSG00000180549	1.594887969	0.045123564
ENSG00000143845	0.705241782	0.045256681
ENSG00000163701	0.885354194	0.046191142
ENSG00000086967	0.812162851	0.046771283
ENSG00000189013	1.800036381	0.046933811
ENSG00000235531	1.11745342	0.047298484
ENSG00000119922	0.721280332	0.047864106

ENSG00000181781	1.517646508	0.049500503
ENSG00000125740	-3.257547079	1.53328E-06
ENSG00000175161	-2.071624331	1.97026E-05
ENSG00000185022	-1.821593264	3.08424E-05
ENSG00000159200	-1.002579722	0.000108023
ENSG00000205085	-0.984622408	0.00012988
ENSG00000198570	-1.503858047	0.000198441
ENSG00000006652	-0.881609917	0.000390914
ENSG00000162892	-3.76275497	0.000340558
ENSG00000183648	-0.834568054	0.000384579
ENSG00000065361	-0.734170375	0.000414932
ENSG00000048162	-0.967347096	0.000538094
ENSG00000109846	-0.858475283	0.000693073
ENSG00000153234	-2.024169309	0.000567011
ENSG00000155893	-1.144178122	0.000498286
ENSG00000142178	-1.098038824	0.000747345
ENSG00000258311	-1.27119987	0.000759129
ENSG00000113739	-1.35464027	0.000829454
ENSG00000116761	-1.69781611	0.000831563
ENSG00000128272	-0.998749847	0.000788554
ENSG00000144655	-0.983291446	0.000849509
ENSG00000146426	-0.999336605	0.000895937
ENSG00000117479	-1.146703408	0.000960259
ENSG00000139998	-0.874418537	0.001030119
ENSG00000170091	-1.763897389	0.00099697
ENSG00000186567	-1.453698983	0.001022831
ENSG00000100146	-1.297320385	0.001169049
ENSG00000152049	-1.045539995	0.001137492
ENSG00000158486	-1.275470432	0.001155092
ENSG00000077044	-1.044446562	0.001333326
ENSG00000173334	-0.897439056	0.001390605
ENSG00000176170	-1.523835434	0.001420175
ENSG00000187527	-2.079093929	0.001526648
ENSG00000116741	-1.049049282	0.00159331
ENSG00000075426	-1.059869953	0.001962178
ENSG00000117472	-1.200587474	0.001759478
ENSG00000124831	-0.744294151	0.002030605
ENSG00000145016	-0.849579111	0.002000921
ENSG00000176697	-1.163340741	0.001972611
ENSG00000185736	-0.820087198	0.001948748
ENSG00000129455	-1.582626606	0.002121407
ENSG00000163873	-0.815170616	0.002137396
ENSG00000258227	-1.367053342	0.002155825
ENSG00000080166	-1.821420839	0.002594732
ENSG00000106366	-1.880860706	0.002514378
ENSG00000157017	-1.000129846	0.002638015
ENSG00000171241	-1.329670105	0.002432021
ENSG00000181649	-1.770052873	0.002582505
ENSG00000197375	-0.767143544	0.00246671
ENSG00000109534	-0.821870473	0.002660799
ENSG00000131127	-0.792439469	0.003222024
ENSG00000143006	-1.382965216	0.003175756

ENSG00000160818	-0.932600235	0.003146832
ENSG00000173826	-1.31502267	0.002735158
ENSG00000184009	-0.840130387	0.002835224
ENSG00000215252	-0.74213865	0.00294548
ENSG00000239672	-0.890333483	0.003264736
ENSG00000174429	-1.409162925	0.003353529
ENSG00000140403	-1.051067585	0.003484327
ENSG00000108846	-1.617720454	0.003772917
ENSG00000119121	-0.881795448	0.003672338
ENSG00000140563	-1.470329502	0.003843803
ENSG00000142871	-1.442922839	0.003701421
ENSG00000103175	-0.851479474	0.004356255
ENSG00000108342	-2.19484782	0.004430251
ENSG00000149300	-1.467070943	0.004149242
ENSG00000161647	-1.028058669	0.004515229
ENSG00000167634	-1.21803393	0.004005208
ENSG00000172020	-1.237687757	0.004416029
ENSG00000172071	-0.900048908	0.004234985
ENSG00000083454	-1.191452097	0.004658286
ENSG00000137709	-0.800013066	0.004695437
ENSG00000140450	-1.063249286	0.004686897
ENSG00000106258	-1.024822595	0.005016107
ENSG00000141655	-0.992423061	0.004937616
ENSG00000156140	-0.905557609	0.004995812
ENSG00000148154	-0.700239934	0.005216136
ENSG00000164761	-1.695190715	0.005225797
ENSG00000178381	-0.778624364	0.005173676
ENSG00000049449	-0.947162077	0.005644812
ENSG00000118193	-0.846823029	0.005628422
ENSG00000119285	-0.714522192	0.00545382
ENSG00000163584	-0.961259238	0.005679052
ENSG00000164611	-1.52965133	0.00579318
ENSG00000184205	-1.026051288	0.005279386
ENSG00000085465	-1.030396218	0.005862897
ENSG00000141378	-0.900622511	0.00618612
ENSG00000021852	-1.441004351	0.006540671
ENSG00000055955	-0.970797748	0.006828266
ENSG00000091127	-0.852624721	0.00736663
ENSG00000120694	-0.762240768	0.006804732
ENSG00000144802	-0.8176701	0.007633833
ENSG00000157119	-0.839487248	0.006339701
ENSG00000163975	-0.921932392	0.006838546
ENSG00000173320	-0.860198651	0.006548968
ENSG00000179165	-1.129063625	0.007508659
ENSG00000180525	-1.106121824	0.007646921
ENSG00000185483	-0.710899201	0.006438191
ENSG00000196872	-0.932796096	0.007186908
ENSG00000197213	-0.730622421	0.008015375
ENSG00000244623	-2.229507314	0.008383172
ENSG00000048462	-0.717370058	0.008480855
ENSG00000188517	-1.177418287	0.008496731
ENSG00000168209	-1.358286363	0.008808175

ENSG00000175264	-1.073381636	0.008839215
ENSG00000184254	-1.156937446	0.00881479
ENSG00000213988	-1.254993975	0.008950735
ENSG00000067082	-0.826502819	0.009071757
ENSG00000115902	-0.998039131	0.009295279
ENSG00000147588	-1.017339135	0.009358436
ENSG00000153922	-0.793322038	0.009283209
ENSG00000071282	-0.753923362	0.009722753
ENSG00000100889	-1.089709958	0.009688149
ENSG00000108821	-0.980130282	0.009872971
ENSG00000248713	-1.195890107	0.00997692
ENSG00000011422	-0.954549278	0.012453972
ENSG00000041982	-1.321881962	0.011979727
ENSG00000113070	-0.809113786	0.012555861
ENSG00000124225	-0.769641727	0.011347766
ENSG00000134668	-1.275641162	0.012041177
ENSG00000141314	-1.114844333	0.012463211
ENSG00000143514	-0.797308605	0.011426404
ENSG00000159166	-1.031210062	0.01082126
ENSG00000166012	-0.827523028	0.011056388
ENSG00000168386	-0.723761007	0.011228973
ENSG00000170561	-1.340320044	0.012337429
ENSG00000171773	-1.381569806	0.012391597
ENSG00000174576	-1.680621817	0.010722651
ENSG00000175894	-1.39922638	0.01125084
ENSG00000182718	-0.701633114	0.011388071
ENSG00000189120	-1.903583817	0.011296842
ENSG00000118985	-1.141349485	0.013584045
ENSG00000144821	-0.867697306	0.01344876
ENSG00000163938	-0.758495221	0.013253934
ENSG00000170961	-0.858439819	0.013562591
ENSG00000256618	-0.960149245	0.013387315
ENSG00000171551	-1.632634521	0.01384253
ENSG00000196083	-0.92681404	0.014141469
ENSG00000115541	-0.722213861	0.015324845
ENSG00000131652	-0.912014312	0.015178508
ENSG00000165732	-0.709568797	0.015475224
ENSG00000172785	-0.768432617	0.01547111
ENSG00000197893	-0.797987367	0.015860055
ENSG00000171476	-1.065272664	0.015953705
ENSG00000166268	-1.397781189	0.01598527
ENSG00000139835	-1.16994372	0.01618297
ENSG00000136997	-1.053418698	0.016341381
ENSG00000183578	-0.967568562	0.016506551
ENSG00000075073	-0.949370345	0.018261302
ENSG00000100003	-0.769100559	0.017799309
ENSG00000102104	-1.048124771	0.01755383
ENSG00000102901	-0.735484654	0.017253626
ENSG00000113916	-0.858878357	0.019141778
ENSG00000118557	-0.75893106	0.018390652
ENSG00000139629	-0.990267621	0.018687203
ENSG00000143320	-0.795653557	0.018065808

ENSG00000148798	-1.684830876	0.018998283
ENSG00000155890	-1.013328187	0.018031359
ENSG00000171121	-0.894780124	0.017396813
ENSG00000197410	-0.890466636	0.01893717
ENSG00000037897	-0.829666335	0.01932832
ENSG00000140798	-1.257791395	0.020205936
ENSG00000123342	-0.865088156	0.020482458
ENSG00000127129	-1.695710725	0.020474433
ENSG00000243709	-1.811246386	0.02050464
ENSG00000100312	-1.382305861	0.02224815
ENSG00000101251	-1.922695005	0.021319821
ENSG00000106868	-0.710063603	0.022939072
ENSG00000122644	-0.845560988	0.021243238
ENSG00000124216	-0.861108939	0.02307002
ENSG00000134259	-0.712446668	0.021352327
ENSG00000143494	-0.770827467	0.022072563
ENSG00000163191	-0.750212984	0.021649785
ENSG00000175766	-1.095566361	0.023131879
ENSG00000178734	-1.022599277	0.022315556
ENSG00000197561	-1.251110872	0.021115385
ENSG00000253320	-0.941996327	0.021326123
ENSG00000186190	-1.240150898	0.02331987
ENSG00000183324	-1.222235377	0.023646518
ENSG00000047662	-0.955031743	0.02382527
ENSG00000135437	-1.153921057	0.023926038
ENSG00000158816	-0.711664221	0.023956013
ENSG00000147174	-0.873245413	0.024403496
ENSG00000154359	-0.715016369	0.025152197
ENSG00000155761	-0.826348036	0.025290819
ENSG00000196517	-1.063552659	0.025324796
ENSG00000170545	-0.715981213	0.025835696
ENSG00000173124	-2.152981471	0.026044127
ENSG00000101680	-0.820867574	0.026201343
ENSG00000105610	-0.810272523	0.026415756
ENSG00000163389	-0.722884689	0.026527389
ENSG00000127507	-0.76020545	0.027084593
ENSG00000137558	-0.992491348	0.026684091
ENSG00000159388	-0.770668473	0.027072534
ENSG00000071539	-0.895062294	0.027484577
ENSG00000122641	-0.871057705	0.027465989
ENSG00000266258	-1.16101567	0.028042671
ENSG00000069696	-1.095967198	0.028330438
ENSG00000092621	-0.77373715	0.028205111
ENSG00000111186	-0.842500648	0.028609285
ENSG00000146955	-0.710832089	0.028286554
ENSG00000171587	-0.702334094	0.028726801
ENSG00000198805	-0.801003231	0.028969372
ENSG00000088726	-0.782684561	0.029338725
ENSG00000162772	-0.929807896	0.029585358
ENSG00000179841	-0.952345291	0.029446884
ENSG00000182308	-0.820897884	0.029678303
ENSG00000115221	-0.749412601	0.030082946

ENSG00000184697	-1.743729616	0.02975076
ENSG00000114013	-0.926994183	0.03044661
ENSG00000160282	-1.029139684	0.030745557
ENSG00000105173	-0.733017204	0.031253521
ENSG00000183117	-1.034226547	0.031231588
ENSG00000092529	-0.776748467	0.031784229
ENSG00000158296	-0.91980045	0.031789458
ENSG00000163817	-1.014456717	0.032957216
ENSG00000181433	-1.314139431	0.033069079
ENSG00000050628	-1.197572581	0.033523116
ENSG00000130649	-0.918999784	0.035140686
ENSG00000151789	-0.713511143	0.03464841
ENSG00000163638	-0.725681651	0.034860153
ENSG00000170522	-0.863516107	0.034167939
ENSG00000172156	-0.937348453	0.033374378
ENSG00000196950	-0.713111197	0.035020771
ENSG00000181026	-0.842337189	0.035434243
ENSG00000103154	-0.897707362	0.040906924
ENSG00000107105	-1.373397852	0.041189784
ENSG00000112494	-2.200510123	0.040830539
ENSG00000112559	-0.722950033	0.039934276
ENSG00000114529	-0.895453473	0.039905517
ENSG00000120937	-1.73760142	0.036741678
ENSG00000125931	-1.127427191	0.037847507
ENSG00000139155	-0.777025669	0.035900795
ENSG00000143867	-1.025474691	0.040506564
ENSG00000144550	-0.753571513	0.038459702
ENSG00000167767	-1.522961424	0.036147245
ENSG00000172482	-1.075938525	0.040619787
ENSG00000221819	-1.30147832	0.038398443
ENSG00000241322	-0.964871953	0.039532095
ENSG00000222005	-1.031887021	0.041930677
ENSG00000086570	-0.841006149	0.042037263
ENSG00000157315	-1.211992919	0.04205383
ENSG00000214827	-0.723477349	0.042331599
ENSG00000179388	-1.035883521	0.043359073
ENSG00000103489	-0.828728804	0.044005725
ENSG00000106211	-0.999925343	0.044536215
ENSG00000115138	-0.794359858	0.045027138
ENSG00000216588	-0.971543147	0.045013087
ENSG00000171495	-1.475510824	0.045895281
ENSG00000102802	-0.725768909	0.046829957
ENSG00000177103	-0.819098614	0.04679255
ENSG00000216895	-1.072213373	0.046738299
ENSG00000145757	-1.241982642	0.048221699
ENSG00000139890	-1.152815745	0.04936772
ENSG00000137648	-1.119332615	0.049708653
ENSG00000196196	-0.719057877	0.049952231

Table S6 Primer information

Gene	Forward	Reverse
Slc19a2	5'-TTGACCGAGAGACAGGTCTTCAA-3'	5'-GACAGGCTTGTAACGGAGGT-3'

Lefty1	5'-TAACAGTGAGCTGGTGCAGG-3'	5'-TGGATGGACACGAGCCTAGA-3'
Bpifb3	5'-TACTGACGGGTTTCTGGCGG-3'	5'-ACTAGGGAGTTCTGGATGGCTTTG-3'
Ecel1	5'-GGCTATGATGACTGGGGAGG-3'	5'-CCTCCCATGTCTGCGATGTT-3'
Trim42	5'-GCCAAGTTCAAAGCAGTCCG-3'	5'-TGGCTCGCAGTTTGAGGAAT-3'
Krt80	5'-CAGCCACCACTACGAGACAT-3'	5'-TCAGCATCCAGGTCCTTCTTC-3'
Rec114	5'-TCGCCCACATGGAAGGTTTTT-3'	5'-CCTGGACGGTGACATACTGG-3'
Mpp3	5'-CGCCGAGGGGAGGTCATA-3'	5'-CAAACCCGAGTCTTCGGACA-3'
Nxnl1	5'-ATGTGTCCCAGGACCCTACA-3'	5'-GCCCGAGGTCTCTCCTCA-3'
Abcc12	5'-TTACAGAAAGGGGTCGTGGC-3'	5'-CCGCTCCCCAATCTCAGTC-3'
Clec5a	5'-AACCACTAATGTGCAGAATGTCTC-3'	5'-ATCCCAGTTGTTGGGACAGG-3'
Tnfrsf11b	5'-CTCACTTGGCCTCCTGCTAA-3'	5'-CTTCGCACAGGGTGACATCTAT-3'
Eif4e1b	5'-TCTACAACCGGGCCAAAGAG-3'	5'-AGACTCCATGTTCCACTGAGAT-3'
Rem2	5'-CCTTGACACCGACATGGACG-3'	5'-TGTCGTATCGGCTTCTGGTGT-3'
Lad1	5'-GCAAGGATGAAGATGCAGACATA-3'	5'-TGACGGATTCTGACCTCTGGA-3'
Npas4	5'-CTGGGCCATTCTATGGTGGA-3'	5'-GTTTGTTGCCTGCACTCTGG-3'
Kcne4	5'-CAAACTGTCAGCCTTTGATCCC-3'	5'-GAGGCTGGACTTCTTCTCCC-3'
Edn2	5'-TCTCCAAAAGCTGAGGGACATC-3'	5'-TCCCAGTAGCTGCCCATTTC-3'
Abcc3	5'-AACTGAGATCCCAAGACCAGC-3'	5'-GTCAGGTTGGAGTCCCAGAA-3'
Myrfl	5'-CGCTCAGATGACACAGGGAT-3'	5'-GCTTTCCTTTGGTAACGCCC-3'
Agxt	5'-TGCAGGCATCGACATCTTGT-3'	5'-TGGGGAATAGAAAAGAACACTCTGG-3'
Tspan1	5'-GTGCAGCAGATCCCGTTCAG-3'	5'-GGCTGCACCACAGAGAAAGATGA-3'
Maff	5'-TTGGGACCACCAGCGACAG-3'	5'-GCTCGCGCTTGACCTTCAG-3'
Hopx	5'-GCTCTCCATCCTTAGCCAGA-3'	5'-TGCTTAAACCATTTCTGCGTCT-3'
Spata9	5'-GGTCAAAATGGCCCTTGCTT-3'	5'-GAACCTTTTCTGACCTGTATGC-3'
Dmrtb1	5'-TTCGCCGACTACGGGCATC-3'	5'-AGTAGGTTGGCTGCAGGTCT-3'
Slc6a20	5'-GTGTATGGGCTGAGGAGATTTG-3'	5'-CGATTCCTGATGAAAGTCCCC-3'
Sphk1	5'-CAGCCCCGAGACTCAAAGAA-3'	5'-GGGGACAGTCTGCTGGTTG-3'
Tmprss4	5'-AGATAGTGGCAGGTGGTAGC-3'	5'-CACTGTCTGGCTCCATATTGC-3'
Fosb	5'-ACCCCGAGAAGAAACACTTACC-3'	5'-ACCCTGGCAAATCTCTCACC-3'
Sel1l2	5'-CTTGGGGATTGCACAGGACA-3'	5'-CAGCCATTTCCATTTCGTGGT-3'
Drd4	5'-CTTTGTCTACTCCGAGGGTGG-3'	5'-ACTGACCCTGCTGGTTGTAG-3'
Abra	5'-CTACGGAGGGGACATGGAGG-3'	5'-TGAGTATCTGTTTGCCTGGGAGTG-3'
Nfic	5'-TTGTGGGATTAGGACGGAGG-3'	5'-CAGGGGACTTCTCACTCGTC-3'
18s	5′-ACCGCAGCTAGGAATAATGGA-3′	5′-GCCTCAGTTCCGAAAACCA-3′

Table S7 *Nfic* shRNA information

shRNA	Forward	Reverse
Nfic shRNA#1	5'-GGTCATGGTCATCCTGTTCAA-3'	5'-TTGAACAGGATGACCATGACC-3'
Nfic shRNA#2	5'-GAGAAGGACTCTACCCAGTAC-3'	5'-GTACTGGGTAGAGTCCTTCTC-3'

Table S8 The CT value of qRT-PCR assay

Gene	CT value of pLko.1	CT value of *Nfic* shRNA
18S	11.08	10.70
Slc19a2	29.60	28.65
Lefty1	NA	NA
Bpifb3	NA	NA
Ecel1	37.19*	37.51*
Trim42	NA	NA
Krt80	29.78	28.67
Rec114	32.53	32.26
Mpp3	39.84*	NA
Nxnl1	36.29*	36.53*

Abcc12	36.55*	38.29*
Clec5a	NA	NA
Tnfrsf11b	27.50	25.98
Eif4e1b	NA	NA
Rem2	36.30*	35.90*
Lad1	38.1*	NA
Npas4	32.39	31.77
Kcne4	36.75*	35.86*
Edn2	36.44*	35.65*
Abcc3	29.98	28.88
Myrfl	36.43*	36.12*
Agxt	NA	NA
Tspan1	33.92	33.73
Maff	30.81	29.12
Hopx	34.26	34.11
Spata9	32.55	32.24
Dmrtb1	34.55	34.09
Slc6a20	36.87*	36.13*
Sphk1	36.04*	35.47*
Tmprss4	35.23*	34.46
Fosb	33.14	31.62
Sel1l2	37.17*	36.94*
Drd4	33.06	32.92
Abra	33.40	33.35

*: When the CT value is greater than 35, we regard it as NA.
